# Ovarian tumors in pediatric age group – A clinicopathologic study of 10 years’ cases in West Bengal, India

**DOI:** 10.4103/0971-5851.71656

**Published:** 2010

**Authors:** Nirmal Kumar Bhattacharyya, Anuradha De, Pranati Bera, Mongal Sristidhar, Subrata Chakraborty, Rajat Bandopadhyay

**Affiliations:** *Department of Pathology and Radiotherapy, North Bengal Medical College, West Bengal, India*

**Keywords:** *Pediatric age group*, *ovarian tumor*, *Germ cell-tumor*, *surface epithelial*

## Abstract

**Background and objectives:**

Objective in this retrospective study is to find out the incidence of different ovarian tumors of girls up to 20 years of age observed in last ten years in North Bengal Medical College and to correlate clinical and gross findings with histopathologic findings and to compare the incidence with other studies and follow-up of patients with malignant ovarian tumors.

**Materials and Methods:**

Findings were retrieved from records of different pathological reports and clinical reports.

**Results:**

Total 151 cases of ovarian tumors were received in pathology department in which 34 cases were malignant (22.6%). Amongst malignant cases, 66% are of germ-cell origin–dysgerminoma being the commonest. Strikingly we got 9 cases of malignant surface epithelial tumor. As per follow-up records most of the dysgerminoma came in stage IA and recovered fully following chemotherapy and radiotherapy. Amongst other malignant tumors, few lost the follow-up management and others expired due to metastasis.

**Conclusions:**

Patients from hilly areas of North Bengal and low socio-economic status led to lower detection rate of ovarian tumors in early stage which are absolutely necessary for proper guidelines of management to reduce mortality.

## INTRODUCTION

The pediatric age group includes adolescence i.e. up to 20 years of age nowadays. Ovarian tumors in children and adolescent girls constitute an important part of gynecological oncology. The commonest gynecologic neoplasm found in the girl-child is of ovarian origin constituting one per cent of all childhood-malignancies and eight per cent of abdominal tumors in children are of ovarian origin. Moreover, 10 to 30% of ovarian neoplasm operated during childhood or adolescent girls are malignant[1] (Behrman RE.2005). Detection of these tumors at such a young age creates much anxiety to the parents and throw a great challenge to all gynecologists or surgeons attending these patients; because in most of the cases, it is difficult to take the decision for appropriate and optimum surgical treatment thus pulling the pathologist into dilemma for diagnosis – as these two aspects have impact for recurrence and prospects of future child-bearing.

It is well-known that germ-cell tumors are the commonest ovarian neoplasm in the first two decades of life constituting approximately two-thirds of all ovarian tumors. Malignant germ cell tumors constitute one-third of germ cell origin tumors and two-thirds of all ovarian malignancy in this age-group[2] (Scully RE *et al*, 1998). Norris and Jensen found that <1% of epithelial carcinoma occurs below 20 years of age[3] (Norris HJ *et al*, 1972). Sex cord stromal tumors are rare tumors accounting for 5%-8% of all ovarian malignancies[4] Berek JS *et al*, 1995). Granulosa cell tumors are found in prepubertal girls in five per cent of cases.

In contrast to past decades, the prognosis of malignant ovarian germ cell tumors have been better because of major developments in the area of management in recent years, but it needs proper histological diagnosis with staging along with tumor-marker estimation and immunohistochemistry-wherever necessary and motivation of patient with proper counseling that increases survival rate.

## AIMS AND OBJECTIVES

In our retrospective analytical study, we wanted to find out the (1) incidence of different ovarian neoplasm in girls below 20 years of age in North Bengal area (2) to correlate histological diagnosis with gross findings and clinical presentations along with follow-ups and (3) to compare the incidence with other studies published in world literature.

## MATERIALS AND METHODS

All ovarian tumor specimens of girls up to 20 years of age sent to the Pathology department from the Gynecolog y department, North Bengal Medical College, West Bengal, in the period from January 1998 to December 2007 i.e. 10 years period were included in the study. Pathology requisition forms accompanying the operated specimens were analyzed in detail with history and operative findings. In many occasions, we had to go through the operative register kept in record sections of the institution. Histopathology reports of each patient were observed carefully, and correlated with gross findings like bilateralism, size, solid, cystic or variegated. Wherever reports of tumors marker were available, the classification of tumors were done along with staging if possible.

## RESULTS

A total of 151 cases of ovarian tumor specimens of ten years period of girls upto 20 years of age were examined.

Table 1 shows the total of 151 specimens of ovarian tumors from girls up to 20 years of age during this ten years period and in them 34 cases were malignant (22.6%). Amongst the 117 cases of benign tumors the mature teratoma is seen in 36 cases (29.5%). Majority of them are surface epithelial tumors like serous cystadenoma, mucinous cystadenoma and endometrotic cyst. Rests are corpus-luteal cysts and follicular cysts. As our cases were divided into four age groups as seen in Table 1, most of the ovarian tumors are seen in the age group from 16 to 20 years (120 cases) i.e. 80%. Only three cases were seen in the age group up to five years of age (2%). Amongst the benign tumors mature teratoma and serous cystadenoma are seen in all age groups. But mucinous tumors are seen beyond 10 years of age. All corpus luteal cysts, endometriotic cysts and most of follicular cysts are seen in age group of 16 to 20 years. One case of Sertoli- Leydig cell tumor was encountered in age group of 11 to 15 years. Amongst the 34 malignant cases, naturally 23 cases are seen in the age group beyond 16 years (69%). Dysgerminoma constitutes nine cases (27%) and is observed in all age groups. Other malignant germ cell tumors like yolk sac tumor, embryonal carcinoma, immature teratoma and mixed germ cell tumors are 13 cases in total, which are seen beyond five years of age. Amongst the malignant cases, nine are surface epithelial origin (three cases of serous cystadenocarcinoma and six cases of mucinous adenocarcinoma) and all of them are seen in age group beyond 16 years. Two cases of Granulosa cell tumors are seen in the latter age group. Overall, the tumors of germ cell origin are 58 in total of which 22 cases are malignant GCT (39%) and amongst all malignant ovarian tumors it constitutes 66%.

**Table 1:** Age group-wise distribution of different ovarian tumors

Age group in yrs	Total no. of cases	Germ cell tumors		Surface epithelial tumors		Sex-cord stromal tumors	Others
		Benign	Malignant	Benign	Malignant		
1 – 5	Benign (2)	Benign cystic teratoma (1)	Dysgerminoma (1)	Serous cyst adenoma (1)			
	Malignant (1)						
6 - 10	Benign (5)	Benign cystic teratoma (1)	Embryonal carcinoma (1)	Serous cyst adenoma (3)			Follicular cyst (1)
	Malignant (1)						
11 -15	Benign (13)	Benign cystic teratoma (5)	Yolk sac tumor (5)	Mucinous cyst adenoma (4)			Corpus luteal cyst(1)
	Malignant (9)		Dysgerminoma (4)	Serous cyst adenoma (3)			
16-20	Benign (97)	Benign cystic teratoma (29)	Dysgerminoma (4)	Mucinous cyst adenoma (23)	Serous adenocarcinoma (3)	Sertoli-Leydig cell tumor (1)	Follicular cyst (4)
	Malignant (23)		Embryonal ca (1)	Serous cyst adenoma (23)	Mucinous adenocarcinoma (6)	Granulosa cell tumor (2)	Corpus luteal cyst (14)
			Mixed GCT (4)				Endometriotic cyst (4)
			Immature teratoma (2)				
Total	Benign (117)	Benign cystic teratoma (36)	Dysgerminoma(9)	Mucinous cyst adenoma (27)	Serous adenocarcinoma (3)	Sertoli-Leydig cell tumor (1)	Follicular cyst (5)
	Malignant (34)		Embryonal ca (2)	Serous cyst adenoma (30)	Mucinous adenocarcinoma (6)	Granulosa cell tumor (2)	Corpus luteal Cyst (15)
			Yolk sac tumor(5)				Endometriotic cyst (4)
			Mixed GCT (4)				
			Immature teratoma (2)				

As seen in Table 2 the clinical features in most cases are vague abdominal pain and mass. Only 30 cases presented with acute pain in abdomen and exploratory laparotomy revealed torsion in 12 Benign cystic teratoma cases and rests are due to chocolate cyst of ovary and hemorrhagic corpus luteal cyst. Anorexia and weight loss were presentation in few cases only (20 cases). Jaundice was seen in only seven cases all of which were malignant ovarian tumors. Two cases of Granulosa cell tumors presented with precocious puberty.

**Table 2:** Modes of presentation

Clinical features	No.of cases
Abdominal swelling and vague pain	113
Acute pain in abdomen	30
Anorexia with weight loss	20
Jaundice	07
Precocious puberty	02

Table 3 shows, the gross and macroscopic features of all ovarian tumors in our cases. The gross findings were divided into three parameters like size of the mass, bilaterality, whether unilocular / multilocular or solid or solid-cystic. Regarding size of the tumors, they are divided into three groups i.e. up to 10 cm, 11 to 15 cm and more them 15 cm. Amongst our 151 cases, only 24 cases were above 15 cm size in which nine were malignant surface epithelial tumors and 15 were benign surface epithelial tumors. Almost all germ cell tumors i.e. 58 cases of germ cell tumors and nine cases of mucinous cystadenoma were between 11 cm to 15 cm size, other ovarian tumors were smaller than 11 cm. Considering the bilateralism, 140 cases were unilateral and 11 were bilateral of which 07 cases were benign cystic teratoma and rests are surface epithelial tumors.

**Table 3:** Gross / Macroscopic findings of ovarian tumors

	No.of cases
Size of the mass	
< 10 cm	− 60
10 – 15 cm	− 67
> 15 cm	− 24
Bilaterality	
Unilateral	− 140
Bilateral	− 11 [Benign cystic teratoma (7), Mucinous cystadenoma (2), Mucinous ca (1), Serous carcinoma (1)]
Cystic / solid	
Unilocular cyst	− 74 (Mostly serous cystadenoma, follicular cyst, C. luteal cyst, endometriotic cyst, benign cystic teratoma)
Multilocular cyst	− 46 (Mucinous cystadenoma, with / without borderline malignancy, benign cystic teratoma)
Solid cystic / variagated	− 31 (Most malignant tumors)

Gross appearance of tumors was important in our study to correlate with histopathological type. Most of our cases were unilocular (74 cases i.e. 49.2%) which include mostly benign cystic teratoma, serous cystadenoma, follicular cyst, corpus-luteal cyst and endometrotic cyst. Multilocular cystic tumors were found in 46 cases of which most are mucinous cystadenoma and borderline mucinous tumors and 16 cases of benign cystic teratoma. Solid or solid cystic / variegated tumors were found in 31 cases and all of them were malignant.

## DISCUSSION

Ovarian tumors in the pediatric age group are not infrequent.[5] Oumachigui *et al*, 1991. found the incidence to be six per cent of all ovarian tumors. Sawai and Sirsat recorded the incidence as 11.2%[6] (Sawai MM *et al*, 1973). Bren and Maxon reported that 35% of all ovarian neoplasms in childhood and adolescent were malignant[7] (Bren JL *et al*, 1977). But we found incidence of malignant ovarian tumors in this age group as 22.6% [Table 1]. Ehren *et al*. found that 60 – 85% of ovarian neoplasm of Pediatric age group were of germ-cell origin[8] (Ehren IM *et al*, 1984). In our study, only 58 cases were of germ cell origin (38%). Amongst malignant tumors 22 cases out of 34 were malignant germ-cell tumors (66%). Dysgerminoma was the commonest malignant GCT (41%).

In our series, it is observed that incidence of germ-cell tumors is slightly lower than the other published reports. On the contrary, there is increased incidence of surface epithelial tumors. It comprises 70 cases out of total 151 cases (i.e. 48%). It includes mostly the benign lesion like serous cystadenoma, mucinous cystadenoma and endometriotic cysts. Malignant lesions within this group are nine in number (27% of malignant ovarian tumors). The incidence of surface epithelial tumors published in most world literature is usually 15 - 20% in the pre pubertal age group and adolescent age group[9] (Graspa D *et al*, 2006). In our series, the rise in incidence is possibly due to the inclusion of cases up to the 20 years of age and a large number of patients have started menstruation with effect of rising sex hormones (estrogen and progesterone) that may a play a role in pathogenesis of such tumors. Other possible causes are that this area is a rural hilly one where rate of illiteracy is higher and as most of germ cell tumors in girl child give vague symptoms, patients are reluctant to attend to such tertiary care hospital resulting in lower incidence of germ cell tumors as compared to other international reports. Karaman A *et al*. in a review article commented that superficial epithelial ovarian tumors are unusual in adolescent girl, extremely rare before menarche[10] (Karaman A *et al*, 2008 Feb.). Only eight cases reported so far in English language and total 14 cases were reported. They have also reported a case of huge mucinous cystadenoma in a 14-year-old girl.

Alobaid AS also reported a huge mucinous cystadenoma of ovary in a 12-year-old girl who presented clinically as bilateral hydronephrosis and renal insufficiency[11] (Alobaid AS, 2008 Jan.).

Regarding gross features in our study cases, 140 cases were unilateral and 11 were bilateral of which most are benign cystic teratoma. So, out of 36 benign cystic teratoma cases, seven were bilateral i.e. 19.4% which are almost similar to other published reports. Solid or solid-cystic ovarian masses are generally indicative of malignancy. We got 31 cases, most of which were malignant, only two cases showing solid-cystic features were of benign (one is dermoid cyst, other is endometriotic cyst). Malignant tumors are mostly germ-cell tumors. In our series we got two cases of granulosa cell tumors and one case of Sertoli-Leydig cell tumor. Of these three cases of sex cord- stromal tumors – only two cases presented with precocious puberty.

As our study is a retrospective one, close follow-up for benign cases was not necessary where involvement was only unilateral. Those benign cases having bilateral tumors had undergone both-sided salpingo-oophorectomy leading to infertility. But patients having malignant ovarian tumors need follow-up regarding appropriate therapy and survival. So far, from the records retrieved from the oncology department of my institute, the 21 patients only of malignant ovarian tumors of this age-group attended post-operatively for radiotherapy and chemo therapy and it is observed that seven out of nine dysgerminoma cases of patients in stage I-A survived till date and other two cases lost to follow-up. Subjects having embryonal carcinoma or other mixed germ tumors were advised appropriate treatment, but most of them lost follow-up and one case who attended in last stage did not survive.

## CONCLUSION

So, in our 10 years’ cases of Pediatric ovarian tumors, we observed the high incidence of malignant tumors. Though the germ cell tumor is the commonest both in benign and malignant groups, there is slightly higher incidence of surface epithelial tumors both in benign and malignant groups as compared to other international reports. Possibility of this higher incidence is inclusion of cases up to 20 years of age in the pediatric age group, where a good number of cases are seen after menarche in which estrogen and progesterone hormone may play a role in pathogenesis of surface epithelial tumors and reluctance of girl children to attend tertiary care hospital with vague symptoms in germ cell tumor due to higher rate of illiteracy in this hilly area.
